# Hernie diaphragmatique post-traumatique

**DOI:** 10.11604/pamj.2015.20.16.5942

**Published:** 2015-01-06

**Authors:** Abdelkarim Shimi, Mohamed Khatouf

**Affiliations:** 1 Service de Réanimation Polyvalente A1, CHU Hassan II, Faculté de Médecine et de Pharmacie, Université Sidi Mohamed Ben Abdellah, Fez, Maroc

**Keywords:** Hernie diaphragmatique post-traumatique, chirurgie traumatique, chirurgie, Post-traumatic diaphragmatic hernia, trauma surgery, surgery

## Image en medicine

L'incidence des ruptures diaphragmatiques chez les patients admis à l'hôpital pour «traumatisme fermé» se situe entre 0,8% et 1,6%. Des lésions associées sont présentes dans 95% -100% des cas. Les ruptures diaphragmatiques surviennent principalement à gauche (68%-84%) et les structures le plus fréquemment herniées sont dans l'ordre décroissant: l'estomac, la rate, le côlon, l'intestin grêle et le foie. Seul 25%-40% des radiographies du thorax réalisées à l'entrée permettent le diagnostic et le CT-scan peut se révéler une aide précieuse. Dans la phase aiguë, ce sont les lésions associées qui déterminent le pronostic. L'approche chirurgicale consiste en une laparotomie ou laparoscopie seule dans la majorité des cas. Nous rapportons le cas d'un patient âgé de 50 ans, admis à notre hôpital pour la prise en charge d'un polytraumatisme, le scanner thoraco-abdominal réalisé dans le cadre du bilan lésionnel a objectivé une hernie diaphragmatique gauche post-traumatique. Le patient était opéré par voie d'abord médiane sus ombilicale, avec découverte d'une brèche diaphragmatique de 7 cm au niveau de la coupole gauche à travers laquelle l'estomac est ascensionné dans l'hémithorax gauche. Le traitement a consisté en une réduction de l'organe hernié avec suture de la brèche diaphragmatique et la mise en place d'un drain thoracique, les suites opératoire étaient simple et le patient a repris conscience a j11 de son admission en réanimation, il a quitté l'hôpital à j18.

**Figure 1:**
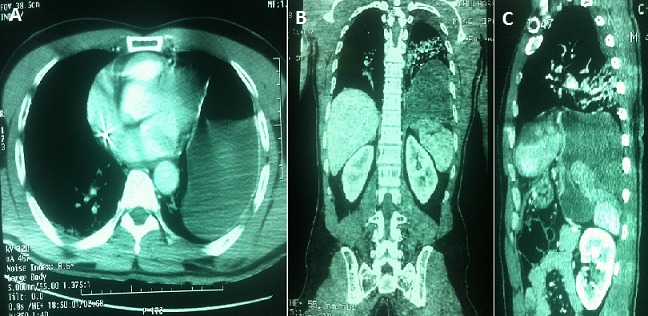
A) scanographique montrant l estomac en intrathoracique; B) reconstitution montrant la perte de continuité diaphragmatique avec la présence de l estomac en sus diaphragmatique; C) reconstruction dans le plan sagittal montrant l estomac hernié en intra-thoracique

